# Protocol of the CONSORT and SPIRIT Extension for multicenter clinical trials

**DOI:** 10.3389/fpubh.2023.1241152

**Published:** 2023-09-15

**Authors:** Xuan Zhang, Chongya Dong, Nana Wang, Chunpong Chan, Chung Tai Lau, Juan Wang, Jiangxia Miao, Chen Yao, Youping Li, Aiping Lyu, David Moher, Zhaoxiang Bian

**Affiliations:** ^1^Chinese Clinical Trial Registry (Hong Kong), Hong Kong Chinese Medicine Clinical Study Centre, Chinese EQUATOR Centre, School of Chinese Medicine, Hong Kong Baptist University, Hong Kong, Hong Kong SAR, China; ^2^Centre for Chinese Herbal Medicine Drug Development, Hong Kong Baptist University, Hong Kong, Hong Kong SAR, China; ^3^Medical Statistics Office, Peking University First Hospital, Beijing, China; ^4^Department of Computer Science, Faculty of Science, Hong Kong Baptist University, Hong Kong, Hong Kong SAR, China; ^5^School of Chinese Medicine, The Chinese University of Hong Kong, Hong Kong, Hong Kong SAR, China; ^6^Chinese Evidence-Based Medicine Center, West China Hospital, Sichuan University, Chengdu, China; ^7^Clinical Epidemiology Program, Ottawa Hospital Research Institute, University of Ottawa, Ottawa, ON, Canada

**Keywords:** center heterogeneity, CONSORT checklist, multicenter clinical trial, reporting guideline, SPIRIT checklist

## Abstract

**Background:**

Multicenter clinical trials play an indispensable role for assessing the efficacy of a new intervention or treatment, particularly in Phase II or III studies. Previous studies have shown that these studies often suffer from inadequate reporting of key details related to their design, implementation, and analysis, both in the protocol and final reports. This limitation reduces the practical and scientific value of the findings. Furthermore, the lack of guidance on how to report multicenter features can contribute to poor reporting. Therefore, this study aims to develop guidelines to improve the reporting of multicenter trials, including two Extensions of the CONSORT 2010 and the SPIRIT 2013.

**Methods/design:**

The standard methodology for developing health research reporting guidelines involves the following steps: (i) Identifying the need for development and launching the research project; (ii) Preparing the registration and reviewing the literatures; (iii) Proposing the initial Checklists and conducting the Delphi exercise; (iv) Arranging the consensus meeting and formulating the Checklists; (v) Conducting the pilot test and drafting explanatory documents (E&E); (vi) Seeking comments from advisory group and finalizing the guidelines; and (vii) Developing the publication and dissemination strategies.

**Conclusion:**

By using the CONSORT and SPIRIT checklists as starting points, the development of extensions specific to multicenter trials can help researchers design and report high-quality clinical research. This, in turn, can facilitate the application of study findings in the current evidence-based healthcare system.

## Introduction

Randomized controlled trials (RCTs) are considered the gold standard for evaluating the effectiveness of a new intervention or treatment (1). Multicenter is a powerful research design that can significantly increase the sample size and improve external validity, making them a common choice for RCTs, particularly in Phase II or III studies (2). As drug development becomes increasingly globalized, multicenter RCTs, especially in international settings, are experiencing rapid growth (3). However, the design and implementation of these studies can be complex and expensive (4). Clear and comprehensive reporting of multicenter trials is crucial for their results to be appropriately included in systematic reviews and practice guidelines, which can lead to better outcomes for routine service and policy-related decisions. Poor reporting of detailed information about multicenter specifics, or a lack of reporting altogether, can weaken the link between research and practice, resulting in the waste of significant resources (5). While poor reporting does not necessarily mean poor methodology or trial conduct, it is only possible to conduct a critical appraisal of trial quality if the study’s design, implementation, and analysis are thoroughly and accurately described in the report (6).

Previous studies have highlighted several problems in multicenter RCTs, including: (i) a lack of criteria for center selection, resulting in poorly performing centers with delayed start-up, unmet target recruitment, and poor data quality, leading to a waste of resources and time (7, 8); (ii) inadequate analysis or adjustment for central effects or heterogeneity (9–12); and (iii) a lack of data management and monitoring, such as the use of central monitoring techniques or on-site monitoring, to ensure data quality across centers (13, 14). In addition, we have investigated the reporting quality of final reports and protocols of multicenter RCTs and have found that the details in multicenter design, implementation, management, analysis, and monitoring are poorly reported, with this suboptimal status persisting over the past few decades (15, 16). As the demand for multicenter studies increases and their results become increasingly valuable to patients and the scientific community, and given the significant expense associated with these trials, improving their reporting should be a priority.

To address these issues, we have proposed extending the Consolidated Standards of Reporting Trials (CONSORT) Statement and the Standard Protocol Items: Recommendations for Interventional Trials (SPIRIT) Statement to address the problems and challenges in multicenter research (17, 18). The development of multicenter studies reporting guidelines will include checklists of reporting items, participant flowcharts (if applicable), and Explanation & Example (E&E) documents that offer authors recommendations to describe these studies accurately, comprehensively, and transparently. The generic guidelines of the SPIRIT 2013 and the CONSORT 2010 have significantly improved the reporting quality of clinical trials, both in protocols and publications (19, 20). Several extensions of them have been developed for different types of study designs, such as SPIRIT for N-of-1 trials (21), SPIRIT for traditional Chinese medicine (22), CONSORT for pragmatic trials (23), and CONSORT for randomized crossover trials (24), etc. However, none of these guidelines can provide direct guidance on reporting multicenter trials, as their details are not covered. Therefore, we propose the development of both the “SPIRIT Extension for Multicenter Clinical Trials Guideline” and “the CONSORT Extension for Multicenter Clinical Trials Guideline” to promote standard reporting. This will facilitate efficient, objective, and accurate transfer of information and findings across various types of multicenter clinical studies.

## Methodologies

**Figure 1:**
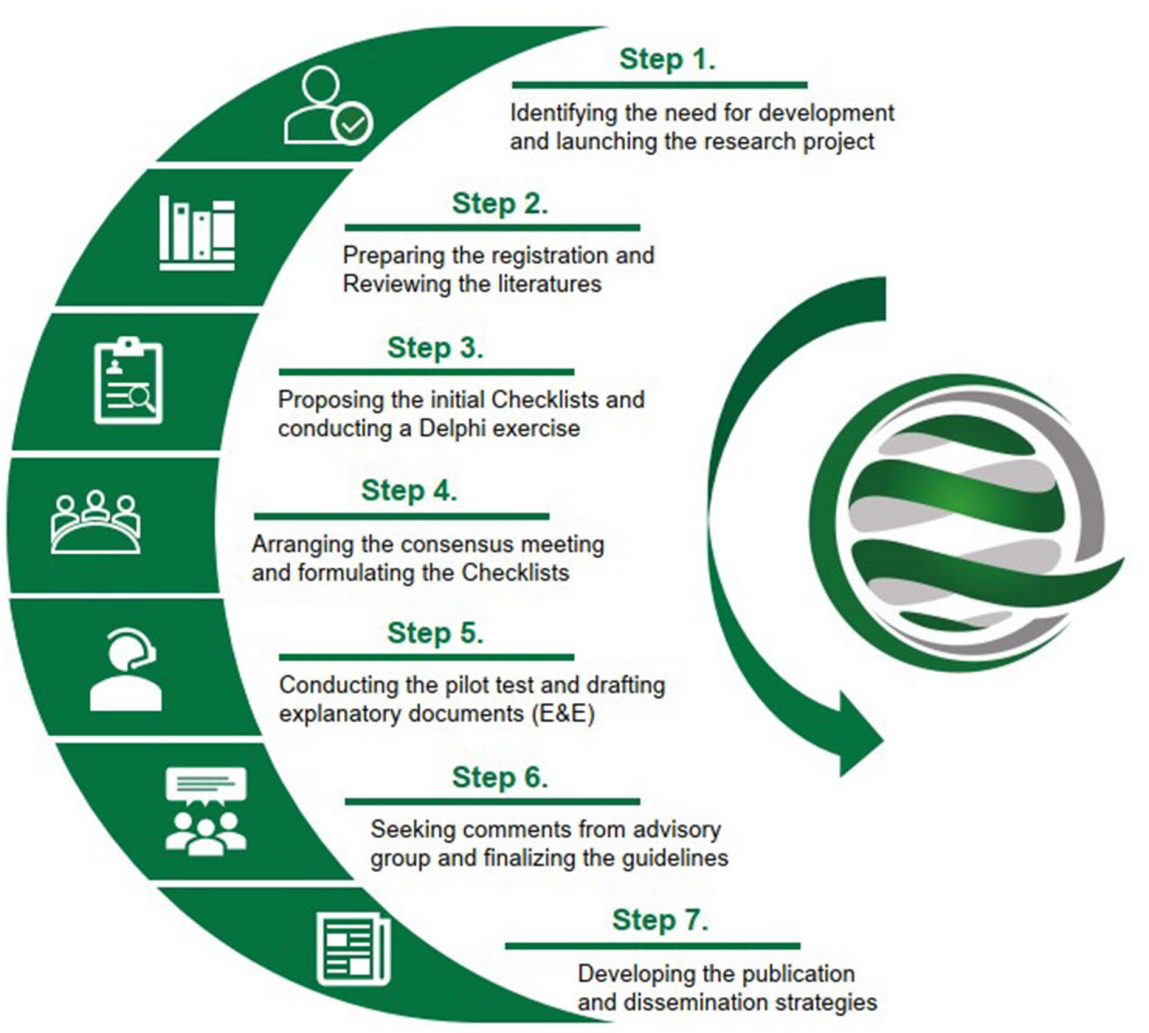
Methodology steps for the development of the CONSORT and SPIRIT Extension for multicenter clinical trials.

Ethical approval for this study was granted by the Research Ethics Committee (REC) of Hong Kong Baptist University (ref: REC/19–20/0493). The development process (Figure 1) will adhere to the methodological framework recommended by the Enhancing the QUAlity and Transparency Of health Research (EQUATOR) Network for developing health reporting guidelines (25). As the consensus process is the core method, each participant in this project will be required to provide consent by accepting our email-based invitation before providing their comments. The invitation letter will be sent from a specific email account and will include an introduction to the project, explanations of the survey or meeting being conducted, and informed consent for participation. Specific steps for the development are listed below:

### Identifying the need for development and launching the research project

Before initiating this research, four authors (CYD, XZ, CY, and ZXB) conducted a literature review of multicenter clinical studies, focusing mainly on review and commentary papers, and discussed the current problems in the reporting and methodology of multicenter designs. This led to the identification of the need for the proposed guidelines (26–28). We then prepared a proposal for funding application, which was subsequently approved. After receiving financial support, we formally launched the project and allocated responsibilities to each group involved in our project (Supplementary material 1).

### Preparing the registration and reviewing the literatures

The working group has registered the proposed two guidelines in the EQUATOR Network (29, 30) and has conducted two literature reviews to assess the reporting quality of multicenter RCTs published as final results or protocols (15, 16). The objectives of the reviews include: (i) summarize the general characteristics of published multicenter RCTs; (ii) assess their reporting quality using the current standard generic guidelines and a special-designed multicenter-related checklist, as well as to analyze any improvements observed after the issuance of CONSORT or SPIRIT guidelines; and (iii) evaluate whether multicenter RCTs, as currently practiced, provide sufficient information to accurately reflect the multicenter design, conduct, and analysis, and identify any common problems. The results provided the basis for drafting the initial items. Additionally, we collected information about the corresponding authors of each included article, as this was useful for identifying Delphi experts.

### Proposing the initial checklists and conducting a Delphi exercise

First, based on the results of the literature reviews, four authors (XZ, CYD, CY, and ZXB) drafted the initial checklists for the CONSORT and the SPIRIT for multicenter RCTs, respectively. Second, we consulted with five experienced guideline developers from Canada, the United Kingdom, and Australia for revisions (Supplementary material 2). Finally, two authors (XZ and CPC) developed web-based Delphi questionnaires accordingly (Supplementary material 3), with one author specializing in computer science and responsible for the maintenance of the online system.

The Delphi method is a structured process used to establish consensus by obtaining information from a group of experts through a series of questionnaires, with each refinement based on feedback from respondents on a previous version. This allows participants to consider the group’s thoughts and to compare and adjust their own assessments in the next round. A strength of this method is that it allows all individuals in a group to communicate their views. Anonymous voting also limits direct confrontation among individuals and the influence of power dynamics and hierarchies on the group’s decision (31–33). In this project, two or three rounds of Delphi exercise are designed to identify the extension items for multicenter trials (Supplementary material 3). In each round of the Delphi survey, participants are asked to rate each item on a scale of “1 (not important)” to “5 (very important),” to suggest new items, and to provide comments; any items that not reach consensus or any new items and comments are circulated in subsequent rounds. Following each round, the score for each item is calculated with the formula of 100% * (1*N_5_ + 0.75*N_4_ + 0.5*N_3_ + 0.25*N_2_) / (N_5_ + N_4_ + N_3_ + N_2_ + N_1_), where N_i_ represents the number of respondents who chose specific “i” in the scale of “1 to 5.” Items with a score greater than or equal to 75% should be included. This calculation formula considers both the consensus level and the weight of responses (34).

Participants invited to the Delphi exercise come from a representative sample of researchers of clinical trials, corresponding authors who have published or registered multicenter clinical studies, methodologists or developers of reporting guidelines, and trialists or potential users such as clinicians, teachers, or graduate students majoring in conventional medicine or complementary alternative medicine (Supplementary material 4). A broad range of disciplines as well as diverse cultures and geographic locations are considered in the selection of participants (35). To ensure an adequate number of respondents (no less than 100), we plan to send at least 200 invitation letters in the first stage, with a response rate estimated at 50%. A supplementary list of potential participants is prepared if the initial response rate is not sufficient. Additionally, no less than three times of reminder emails are ensured during the validity period to improve the response rate. Anonymity and confidentiality of responses are ensured, and the collection and analysis of data are authorized only to the working group members.

### Arranging the consensus meeting and formulating the checklists

We place a high preference on conducting a face-to-face consensus meeting as an essential step in formulating the guideline, regardless of whether it is conducted online or offline. We will invite professionals with expertise relevant to clinical trial methodology, clinical epidemiology, evidence-based medicine, multicenter study design and implementation, as well as several experts who have participated in the development of reporting guidelines. The number of participants is expected to be around 11–15. Initially, we will identify a small number of invitees whose participation we consider essential to the meeting. Once they accept our invitation, we will set the meeting date and invite the remaining participants. If necessary, we will consider a supplementary list of invited experts. At least one week before the meeting date, we will send the meeting agenda and research materials (such as the consent form, previous results, and the two checklists) to the participants.

During the meeting, the project leader will introduce each participant and invite a meeting chair. Working group members will give a presentation focusing on the results of the Delphi survey. In regard to the excluded items in the Delphi process, all participants will actively engage in a focused discussion and anonymously vote on the inclusion of each item. If the meeting is conducted offline, participants will be requested to record their choices on paper without providing their signature. On the other hand, if the meeting takes place online, the Zoom voting function will be utilized. This will enable participants to indicate their agreement, disagreement, or abstention from voting regarding the items. The strength of the consensus will be judged using the following scale: (1) Simple Majority – No Consensus (50.1–59% agreement), (2) Majority – Weak Consensus (60–65% agreement), (3) Super Majority – Strong Consensus (66–99% agreement), and (4) Unanimous – 100% agreement (36). After voting on each item, we will present the aggregated results to all experts for confirmation.

In addition, the following items will be included in the meeting: (i) Review all included items in the Delphi for finalization; (ii) Discuss the possibility of developing a diagram for multicenter trial specifics and, if appropriate, consider its content; (iii) Discuss strategies for producing documents, identify who will be involved in which activities (such as guideline statement, explanatory documents, and example collection); discuss authorship if applicable; and (iv) Discuss a dissemination strategy, such as publication, translation, and endorsement of journals if time permits. At the end of the meeting, each expert will be asked to review the checklists again to confirm that their comments were appropriately understood and considered. After the meeting, experts will receive the formulated checklists for signature.

### Conducting the pilot test and drafting explanatory documents (E&E)

To identify practical challenges with any of the included items, pilot testing before or after the consensus meeting will be considered, if applicable. The potential users for testing will include: (i) Graduate students who have studied a clinical trial methodology course in universities; (ii) Authors (such as the first author and/or corresponding author) who have published or registered multicenter RCTs, particularly in recent years; (iii) Researchers or staff from major pharmaceutical companies with experience in conducting multicenter trials. These targeted individuals will be contacted by email (or telephone, if necessary) to obtain their comments on the utility and clarity of the items. Feedback from the pilot test will be used to refine the wording and presentation of the checklist. Useful comments will be incorporated into the E&E documents.

Based on the above results, our working group will prepare the guideline statements and E&E documents. The main contents will include: (i) A short document of approximately 3,000–4,000 words reporting on the rationale for developing the guideline and the development process; (ii) The checklist tables, which will include the original CONSORT or SPIRIT items, relevant extensions (and new additions) for multicenter RCT specifics, as well as the modified flow diagram (if any); (iii) A detailed justification and explanation of each item in the reporting checklists. The explanatory document will be considerably longer than the guideline statement and will mainly include the rationale for extensions, characteristics of multicenter studies, and the necessity for reporting. Relevant thematic concepts could be reported in Supplementary material; (iv) An example document for good reporting. It is essential to demonstrate how the extension items are well presented. Thus, each extension item will include one or more examples of good reporting from published multicenter RCTs. All of the above materials will be organized and included in the manuscripts, and the drafted manuscripts will be forwarded to advisory experts for additional comments and revisions.

### Seeking comments from advisory group and finalizing the guidelines

We will consult with advisors for the revision and finalization of the guidelines with E&E documents. An advisory group will be established, comprising of 5–7 experts with international reputations from different areas of expertise, including policymakers, methodologists, and editors, among others. At least 1/3 of the members with experience in guideline development will be ensured. Based on the comments from the advisory group, further revisions of the manuscripts will be conducted by the working group members. Then, each author of the manuscripts will review the revisions and provide approval of the final version.

### Developing the publication and dissemination strategies

To facilitate the dissemination of the guidelines, we will develop publication and promotion strategies. Firstly, we have registered the guidelines in the EQUATOR Network and will keep all relevant information updated. In addition, a dedicated webpage of the Chinese EQUATOR Center^1^ will be used to discuss new, relevant evidence related to multicenter trials, and to ask the wider scientific community to provide feedback on their experiences of using the guideline, allowing for the guideline’s continual development. Secondly, simultaneous publications in high-impact journals (preferably open access) will be conducted. Moreover, Chinese versions of the guidelines (according to the CONSORT translation policy) will be considered to facilitate broad dissemination. Thirdly, the working group members will present the guidelines at influential conferences, professional bodies, and organizations within their respective fields. Finally, we will use information technology to raise awareness throughout the study, such as social media platforms like Facebook, Twitter, and LinkedIn. We will also invite editors from relevant journals to endorse the guidelines. If applicable, we would like to explore collaborations with the Chinese Clinical Trial Registration and other clinical trial registries within the World Health Organization International Clinical Trials Registry Platform (WHO ICTRP) to have our Guidelines included on their websites. This would provide a significant opportunity to reach a larger audience of our target users. By making the Guidelines accessible on these platforms, the chances of them being widely recognized and utilized would greatly increase.

## Discussion

High-quality multicenter RCTs can strengthen the external validity of research findings in practice, accelerate drug development, and promote more efficient translation of scientific knowledge. As a result, the number of multicenter trial publications has significantly increased in recent decades. Ensuring that multicenter research is complete, accurate, transparent, and timely is a shared responsibility of all stakeholders involved in the healthcare system. The development of the CONSORT and SPIRIT extensions for multicenter clinical studies aims to identify key information to be included in protocols and final reports, thereby improving the quality of trial design, implementation, and reporting. These extensions can be adopted in conjunction with the original SPIRIT and CONSORT statements, as well as other extensions, to account for variations in study methodology, including different design aspects (such as pragmatic trials), interventions (such as non-pharmacologic treatments), outcome measures, and data (such as patient-reported outcomes). We hope that the CONSORT and SPIRIT extensions for multicenter clinical trials will further facilitate the application of study findings in the current evidence-based healthcare system. A timeline outlining the implementation of the study is provided in Supplementary material 5, which aligns with the methodology steps described in this protocol. This timeline serves as a guide for the sequential execution of various activities and milestones during the study. By following this timeline, researchers can ensure a systematic and organized approach to the implementation of the study, adhering to the prescribed methodology outlined in the protocol.

## Ethics statement

The conduct of this research project will conform to the appropriate ethical and legal standards regarding informed consent, confidentiality, and data storage. Ethics approval was obtained from the Research Ethics Committee (REC) of Hong Kong Baptist University (ref: REC/19-20/0493).

## Author contributions

ZB and DM conceived this study. ZB and XZ designed the project, drafted, revised, and finalized the manuscript. ZB, CY, CD, and XZ drafted the initial checklists. DM, YL, and ZB revised and finalized the initial checklists. XZ, CC, NW, CL, JW, and ZB conducted and summarized the results of literature reviews and the Delphi survey. JM, CY, YL, AL, DM, and ZB provided critical comments for this protocol. All authors approved the final version of the manuscript.

## Funding

This work was supported by the Health and Medical Research Fund (Ref: 18192671), the Food and Health Bureau, The Government of the Hong Kong Special Administrative Region, Hong Kong, China; Chinese Medicine Development Fund (No. 20B2/027A), Hong Kong, China; China Center for Evidence Based Traditional Chinese Medicine, CCEBTM (2020YJSZX-5); and Health@InnoHK Initiative Fund of the Hong Kong Special Administrative Region Government (ITC RC/IHK/4/7). The funders had no role in the design of the study, in the collection, analysis, and interpretation of data, nor in the writing of the manuscript.

## Conflict of interest

The authors declare that the research was conducted in the absence of any commercial or financial relationships that could be construed as a potential conflict of interest.

## Publisher’s note

All claims expressed in this article are solely those of the authors and do not necessarily represent those of their affiliated organizations, or those of the publisher, the editors and the reviewers. Any product that may be evaluated in this article, or claim that may be made by its manufacturer, is not guaranteed or endorsed by the publisher.

## Footnotes

^1^ http://www.equator-chinese.org/
