# Supplementary material for: Protocol of the CONSORT and SPIRIT Extension for multicenter clinical trials
Source: Front Public Health. 2023 Sep 15;11:1241152. doi: 10.3389/fpubh.2023.1241152 (PMC10540686; doi:10.3389/fpubh.2023.1241152)
Supplement: Supplementary file 1 [file Data_Sheet_1.PDF]

Supplementary information

CONTENT

S1. Responsibilities of each group in the project. .... **- 1 -**

S2. Initial Checklists of the guidelines. .... **- 2 -**

S3. Web-based Delphi questionnaires. .... **- 13 -**

S4. Characteristics of participants in the Delphi survey. .... **- 15 -**

S5. The timeline for the implementation of this study. .... **- 20 -**

S1. Responsibilities of each group in the project.

Group	Responsibility
Working Group	1) Project design and registration; 2) Drafting the proposal and applying the grant; 3) Conducting literature review and proposing initial Checklist; 4) Conducting the Delphi survey and analyzing the results; 5) Organizing and conducting the consensus meeting; 6) Seeking feedback from pilot test; 7) Preparing and finalizing the manuscript; and 8) Promoting the dissemination and updating the reporting guideline.
Delphi Experts Group	1) Providing scoring and comments for all proposed extension items; 2) Deciding which items should be included and excluded (participate in two or three rounds of the Delphi exercises); 3) Suggesting new items which should be added; 4) Reviewing the results of each round of Delphi survey.
Consensus Meeting Group	1) Discussing and refining for each item based on the Delphi results; 3) Voting about the inclusion of each item and deciding the precise wording; 4) Finalizing and signing the consensus-based Checklist; 5) Providing comments for the E&E contents, if applicable.
Advisory Group	1) Reviewing and providing comments for the Checklists; 2) Commenting the guideline manuscripts; 3) Suggesting the strategies for dissemination.

S2. Initial Checklists of the guidelines.

Table 2.1 Initial Checklist of the CONSORT for Multicenter Clinical Trials

Section/Topic	Item Number	CONSORT Checklist Item	Extensions
**Title and abstract**	1a	Identification as a randomised trial in the title.	*Identification as a multicentre trial in the title.*
	1b	Structured summary of trial design, methods, results, and conclusions (for specific guidance see CONSORT for abstracts45 65).	
	Trial design	Description of the trial design (e.g. parallel, cluster, non-inferiority).	*Description of the multicentre trial design.*
	Methods		
	Participants	Eligibility criteria for participants and the settings where the data were collected.	
	Interventions	Interventions intended for each group.	
	Objective	Specific objective or hypothesis.	
	Outcome	Clearly defined primary outcome for this report.	
	Randomization	How participants were allocated to interventions.	*Statement of whether central randomisation was used.*
	Blinding (masking)	Whether or not participants, care givers, and those assessing the outcomes were blinded to group assignment.	
	Results		
	Numbers randomized	Number of participants randomized to each group.	
	Recruitment	Trial status.	
	Numbers analysed	Number of participants analysed in each group.	
	Outcome	For the primary outcome, a result for each group and the estimated effect size and its precision.	*Statement of whether heterogeneity of outcomes across centres was identified in the primary outcomes, and of whether the original inference was robust regarding heterogeneity.*
	Harms	Important adverse events or side effects.	

	Conclusions	General interpretation of the results.	
	Trial registration	Registration number and name of trial register.	
	Funding	Source of funding.	
**Introduction**			
Background and objectives	2a	Scientific background and explanation of rationale.	
	2b	Specific objectives or hypotheses.	
**Methods**			
Trial design	3a	Description of trial design (such as parallel, factorial) including allocation ratio.	
	3b	Important changes to methods after trial commencement (such as eligibility criteria), with reasons.	
Participants	4a	Eligibility criteria for participants.	
	4b	Settings and locations where the data were collected.	
	Add 4c		*Description of any methods used to ensure that participants' recruitment and inclusion was consistent across centres.*
Interventions	5	The interventions for each group with sufficient details to allow replication, including how and when they were actually administered.	*Statement of whether implementation of the interventions was the same across centres. Specify and give the rationale for any disparity.*
Outcomes	6a	Completely defined pre-specified primary and secondary outcome measures, including how and when they were assessed.	
	6b	Any changes to trial outcomes after the trial commenced, with reasons.	
Sample size	7a	How sample size was determined.	
	7b	When applicable, explanation of any interim analyses and stopping guidelines.	
Randomisation:			
Sequence generation	8a	Method used to generate the random allocation sequence.	

	8b	Type of randomisation; details of any restriction (such as blocking and block size).	*Details of stratification or matching if used as part of multicentre design.*
Allocation concealment mechanism	9	Mechanism used to implement the random allocation sequence (such as sequentially numbered containers), describing any steps taken to conceal the sequence until interventions were assigned.	*Details of the implementation procedure of concealment methods among different centres.*
Implementation	10	Who generated the random allocation sequence, who enrolled participants, and who assigned participants to interventions.	*Specification of the procedure for centralised randomisation. If randomisation was not centralised, report how its implementation varied among centres.*
Blinding	11a	If done, who was blinded after assignment to interventions (for example, participants, care providers, those assessing outcomes) and how.	
	11b	If relevant, description of the similarity of interventions	
Statistical methods	12a	Statistical methods used to compare groups for primary and secondary outcomes.	
	12b	Methods for additional analyses, such as subgroup analyses and adjusted analyses.	*Statistical methods for assessment of the heterogeneity of treatment effects across centres and adjustment for centre effect.*
**Results**			
Participant flow (a diagram is strongly recommended)	13a	For each group, the numbers of participants who were randomly assigned, received intended treatment, and were analysed for the primary outcome.	*Report, for each centre, the participant flow through stages of randomised assignment, treatment received, and outcome analysed.*
	13b	For each group, losses and exclusions after randomisation, together with reasons.	*Report, for each centre, losses and exclusions of participants after randomisation, with reasons.*
Recruitment	14a	Dates defining the periods of recruitment and follow-up.	*Centre-based recruitment information, including recruitment strategies and the defining period of recruitment across the included centres.*
	14b	Why the trial ended or was stopped.	
Baseline data	15	A table showing baseline demographic and clinical characteristics for each group.	*A table showing baseline demographic and clinical characteristics for each group and centre.*

Numbers analysed	16	For each group, number of participants (denominator) included in each analysis and whether the analysis was by original assigned groups.	*Report numbers analysed in each centre.*
Outcomes and estimation	17a	For each primary and secondary outcome, results for each group, and the estimated effect size and its precision (such as 95% confidence interval).	*Report centre-based primary outcome results or important secondary outcomes for each group, including the estimated effect size and its precision (such as 95% confidence interval).*
	17b	For binary outcomes, presentation of both absolute and relative effect sizes is recommended.	
Ancillary analyses	18	Results of any other analyses performed, including subgroup analyses and adjusted analyses, distinguishing pre-specified from exploratory.	*Heterogeneity assessment of treatment effects across centres and adjustment for centre effect, and its justification, if any.*
Harms	19	All important harms or unintended effects in each group (for specific guidance see CONSORT for harms42).	
**Discussion**			
Limitations	20	Trial limitations, addressing sources of potential bias, imprecision, and, if relevant, multiplicity of analyses.	*Interpretation of potential bias among different centres, if any.*
Generalisability	21	Generalisability (external validity, applicability) of the trial findings.	
Interpretation	22	Interpretation consistent with results, balancing benefits and harms, and considering other relevant evidence.	
Other information			
Registration	23	Registration number and name of trial registry.	
Protocol	24	Where the full trial protocol can be accessed, if available.	
Funding	25	Sources of funding and other support (such as supply of drugs), role of funders.	

Table 2.2 Initial Checklist of the SPIRIT for Multicenter Clinical Trials

Section/item	Item Number	SPIRIT checklist items	Extensions
**Administrative information**			
Title	1	Descriptive title identifying the study design, population, interventions, and, if applicable, trial acronym.	*Identification as a multicentre trial design in the title.*
Trial registration	2a	Trial identifier and registry name. If not yet registered, name of intended registry.	
	2b	All items from the World Health Organization Trial Registration Data Set.	
Protocol version	3	Date and version identifier	
Funding	4	Sources and types of financial, material, and other support.	*If the funding(s) are supported by different centres, please specify.*
Roles and responsibilities	5a	Names, affiliations, and roles of protocol contributors.	
	5b	Name and contact information for the trial sponsor.	*Name and contact information of lead investigator(s) for each participating centre, if applicable (provide in supplementary material).*
	5c	Role of study sponsor and funders, if any, in study design; collection, management, analysis, and interpretation of data; writing of the report; and the decision to submit the report for publication, including whether they will have ultimate authority over any of these activities.	
	5d	Composition, roles, and responsibilities of the coordinating	*Composition, roles, and responsibilities of each*

		centre, steering committee, endpoint adjudication committee, data management team, and other individuals or groups overseeing the trial, if applicable (see Item 21a for data monitoring committee).	*participating centre, if applicable (provide in supplementary material).*
**Introduction**			
Background and rationale	6a	Description of research question and justification for undertaking the trial, including summary of relevant studies (published and unpublished) examining benefits and harms for each intervention.	*Rationale for using a multicentre design.*
	6b	Explanation for choice of comparators.	
Objectives	7	Specific objectives or hypotheses.	
Trial design	8	Description of trial design including type of trial (eg, parallel group, crossover, factorial, single group), allocation ratio, and framework (eg, superiority, equivalence, noninferiority, exploratory).	*Description of the multicentre trial design.*
**Methods: Participants, interventions, and outcomes**			
Study setting	9	Description of study settings (eg, community clinic, academic hospital) and list of countries where data will be collected. Reference to where list of study sites can be obtained.	*Description of each participating centre.*
Eligibility criteria	10	Inclusion and exclusion criteria for participants. If applicable, eligibility criteria for study centres and individuals who will perform the interventions (eg, surgeons, psychotherapists).	*Eligibility criteria for participating centre. If applicable, provide the rationale for criteria.*
Interventions	11a	Interventions for each group with sufficient detail to allow replication, including how and when they will be administered.	*State whether interventions will be administered the same across centres.*

	11b	Criteria for discontinuing or modifying allocated interventions for a given trial participant (eg, drug dose change in response to harms, participant request, or improving/worsening disease).	*Criteria for any potential disparity of allocated interventions maybe existed in the actual implementation across centres, if applicable.*
	11c	Strategies to improve adherence to intervention protocols, and any procedures for monitoring adherence (eg, drug tablet return, laboratory tests).	*Description of methods used to ensure that interventions will be administered consistently across centres.*
	11d	Relevant concomitant care and interventions that are permitted or prohibited during the trial.	
Outcomes	12	Primary, secondary, and other outcomes, including the specific measurement variable (eg, systolic blood pressure), analysis metric (eg, change from baseline, final value, time to event), method of aggregation (eg, median, proportion), and time point for each outcome. Explanation of the clinical relevance of chosen efficacy and harm outcomes is strongly recommended.	*Description of any method(s) to ensure consistent outcome measurement across centres.*
Participant timeline	13	Time schedule of enrolment, interventions (including any run-ins and washouts), assessments, and visits for participants. A schematic diagram is highly recommended (see Figure).	*List the time schedule of different centres if varied.*
Sample size	14	Estimated number of participants needed to achieve study objectives and how it was determined, including clinical and statistical assumptions supporting any sample size calculations.	*Specification of sample size pre-allocation among study centres, if any.*
Recruitment	15	Strategies for achieving adequate participant enrolment to reach target sample size.	*Description of the recruitment strategies across centres, if varied.*
**Methods: Assignment of interventions (for controlled trials)**			
Allocation:<br>Sequence generation			
	16a	Method of generating the allocation sequence (eg, computer-	*Statement of whether allocation sequence will be*

		generated random numbers), and list of any factors for stratification. To reduce predictability of a random sequence, details of any planned restriction (eg, blocking) should be provided in a separate document that is unavailable to those who enrol participants or assign interventions.	*stratified by centre or will be centralized.*
Allocation concealment mechanism	16b	Mechanism of implementing the allocation sequence (eg, central telephone; sequentially numbered, opaque, sealed envelopes), describing any steps to conceal the sequence until interventions are assigned.	*State whether the allocation concealment mechanism will be the same in all centres, if not, describe the differences.*
Implementation	16c	Who will generate the allocation sequence, who will enrol participants, and who will assign participants to interventions.	*State whether central randomization will be used. If not, report how its randomization will be implemented among centres.*
Blinding (masking)	17a	Who will be blinded after assignment to interventions (eg, trial participants, care providers, outcome assessors, data analysts), and how.	
	17b	If blinded, circumstances under which unblinding is permissible, and procedure for revealing a participant's allocated intervention during the trial.	
**Methods: Data collection, management, and analysis**			
Data collection methods	18a	Plans for assessment and collection of outcome, baseline, and other trial data, including any related processes to promote data quality (eg, duplicate measurements, training of assessors) and a description of study instruments (eg, questionnaires, laboratory tests) along with their reliability and validity, if known. Reference to where data collection forms can be found, if not in the protocol.	*Description of any central system that will be used to facilitate data collection, analysis, and monitoring among centres.*

	18b	Plans to promote participant retention and complete follow-up, including list of any outcome data to be collected for participants who discontinue or deviate from intervention protocols.	
Data management	19	Plans for data entry, coding, security, and storage, including any related processes to promote data quality (eg, double data entry; range checks for data values). Reference to where details of data management procedures can be found, if not in the protocol.	*Description of data management plan across centres, if this will vary.*
Statistical methods	20a	Statistical methods for analysing primary and secondary outcomes. Reference to where other details of the statistical analysis plan can be found, if not in the protocol.	
	20b	Methods for any additional analyses (eg, subgroup and adjusted analyses).	*Statistical methods for assessment of the heterogeneity of treatment effects across centres and adjustment for centre effect.*
	20c	Definition of analysis population relating to protocol non-adherence (eg, as randomised analysis), and any statistical methods to handle missing data (eg, multiple imputation).	
**Methods: Monitoring**			
Data monitoring	21a	Composition of data monitoring committee (DMC); summary of its role and reporting structure; statement of whether it is independent from the sponsor and competing interests; and reference to where further details about its charter can be found, if not in the protocol. Alternatively, an explanation of why a DMC is not needed.	*Description of data monitoring methods across centres, if this will vary.*
	21b	Description of any interim analyses and stopping guidelines, including who will have access to these interim results and make the final decision to terminate the trial.	*Description of any monitor criteria for centre(s) changed during the trial.*

Harms	22	Plans for collecting, assessing, reporting, and managing solicited and spontaneously reported adverse events and other unintended effects of trial interventions or trial conduct.	*Adverse events and other unintended effects from each center, if any.*
Auditing	23	Frequency and procedures for auditing trial conduct, if any, and whether the process will be independent from investigators and the sponsor.	*Statement of whether auditing will be conducted for each centre, as well as centrally.*
**Ethics and dissemination**			
Research ethics approval	24	Plans for seeking research ethics committee/institutional review board (REC/IRB) approval.	*Statement of whether ethics approval will be conducted by local RECs/IRBs or/and a central REC/IRB.*
Protocol amendments	25	Plans for communicating important protocol modifications (e.g., changes to eligibility criteria, outcomes, analyses) to relevant parties (e.g., investigators, REC/IRBs, trial participants, trial registries, journals, regulators).	
Consent or assent	26a	Who will obtain informed consent or assent from potential trial participants or authorised surrogates, and how (see Item 32).	*Description of any variation among procedures and requirements for informed consent at different centres, particularly for multinational trials.*
	26b	Additional consent provisions for collection and use of participant data and biological specimens in ancillary studies, if applicable.	
Confidentiality	27	How personal information about potential and enrolled participants will be collected, shared, and maintained in order to protect confidentiality before, during, and after the trial.	*Statement of whether each centre will handle personal information in the same way, and of whether all centres will implement the data sharing statement.*

Declaration of interests	28	Financial and other competing interests for principal investigators for the overall trial and each study site.	
Access to data	29	Statement of who will have access to the final trial dataset, and disclosure of contractual agreements that limit such access for investigators.	*Statement of whether final trial dataset/centre data will be accessible to researchers in each centre.*
Ancillary and post-trial care	30	Provisions, if any, for ancillary and post-trial care, and for compensation to those who suffer harm from trial participation.	
Dissemination policy	31a	Plans for investigators and sponsor to communicate trial results to participants, healthcare professionals, the public, and other relevant groups (eg, via publication, reporting in results databases, or other data sharing arrangements), including any publication restrictions.	
	31b	Authorship eligibility guidelines and any intended use of professional writers.	
	31c	Plans, if any, for granting public access to the full protocol, participant-level dataset, and statistical code.	
**Appendices**			
Informed consent materials	32	Model consent form and other related documentation given to participants and authorised surrogates.	*If different forms will be used in different centres, provide the sample(s).*
Biological specimens	33	Plans for collection, laboratory evaluation, and storage of biological specimens for genetic or molecular analysis in the current trial and for future use in ancillary studies, if applicable.	*Description of how procedures for collecting biological specimens will be coordinated across centres.*

S3. Web-based Delphi questionnaires.

S3.1 QR codes of the Delphi online survey of the CONSORT for Multicenter Clinical Trials

CONSORT Extension for
Multicenter Clinical Trials
(1st Round for Delphi Survey)

CONSORT Extension for
Multicenter Clinical Trials
(2nd Round for Delphi Survey)

CONSORT Extension for
Multicenter Clinical Trials
(3rd Round for Delphi Survey)

S3.2 QR codes of the Delphi online survey of the SPIRIT for Multicenter Clinical Trials

SPIRIT Extension for
Multicenter Clinical Trials
(1st Round for Delphi Survey)

SPIRIT Extension for
Multicenter Clinical Trials
(2nd Round for Delphi Survey)

S4. Characteristics of participants in the Delphi survey.

Table 4.1 Characteristics of 153 participants in the Delphi survey of the CONSORT for Multicenter Clinical Trials

Category	Number (n=153, %)
**Gender**	
Male	84 (54.9)
Female	69 (45.1)
**Working experience (Years)**	
≤10	50 (32.7)
10-20	47 (30.7)
20-30	29 (19.0)
>30	27 (17.6)
**Professional field***	
Clinical practice in Western medicine	9 (5.9)
Clinical practice in Traditional Medicine	63 (41.2)
Clinical research methodology	35 (22.9)
Development of reporting guideline	12 (7.8)
Statistical analysis	9 (5.9)
Epidemiology	10 (6.5)
Editor of medical journal	5 (3.3)
Others[1]	10 (6.5)
**Professional title***	
Professor/Clinical Professor, or above	56 (36.6)
Associate Professor/Associate Clinical Professor, or relevant	24 (15.7)
Assistant Professor/Assistant Clinical Professor, or relevant	19 (12.4)
Lecturer, or relevant	17 (11.1)
Postdoc Fellow, or relevant	5 (3.3)
Research Assistant, or relevant	16 (10.5)

PhD Students	11	(7.2)
Others[2]	5	(3.3)
**Types of Institutions***		
University	91	(59.5)
Hospital	34	(22.2)
Research institute	18	(11.8)
Journal agency	4	(2.6)
Government	2	(1.3)
Industry	1	(0.7)
Individual	1	(0.7)
Others[3]	2	(1.3)
**Geographical distribution**		
Mainland of China	72	(47.1)
HKSAR, China	20	(13.1)
Australia	11	(7.2)
South Korea	10	(6.5)
UK	9	(5.9)
US	5	(3.3)
Canada	4	(2.6)
Netherlands	3	(2.0)
Japan	3	(2.0)
Germany	2	(1.3)
India	2	(1.3)
Switzerland	2	(1.3)
Norway	1	(0.7)
Brazil	1	(0.7)
Cyprus	1	(0.7)
Egypt	1	(0.7)

France	1 (0.7)
Italy	1 (0.7)
Norway	1 (0.7)
Serbia	1 (0.7)
Sweden	1 (0.7)
Thailand	1 (0.7)
Uganda	1 (0.7)

* Multiple choices for each participant, thus the total number is more than 153.

1 Including Integrative health scientist (1), Clinical researcher (1), Pharmacologist (1), Clinical dietetics (1), Integrative Chinese and western clinicians and researchers (2), Clinical Pharmacology (1), Clinical research in traditional medicine (1), Global health and health system (1), Clinical research in Western Medicine (1).

2 Including Editor (2), House physician (1), Research manager (1), Not specified (1).

3 Including Not specified (2).

Table 4.2 Characteristics of 124 participants in the Delphi survey of the SPIRIT for Multicenter Clinical Trials

Category	Number (n=124, %)
**Gender**	
Male	68 (54.8)
Female	56 (45.2)
**Working experience (Years)**	
≤10	36 (29.0)
10-20	41 (33.1)
20-30	20 (16.1)
>30	27 (21.8)
**Professional field***	
Clinical practice in Traditional Medicine	75 (60.5)
Clinical practice in Western medicine	9 (7.3)
Methodology and Guideline development	35 (28.2)

Editor of medical journal	12	(9.7)
Clinical trial research (investigator/coordinator)	27	(21.8)
Epidemiology	12	(9.7)
Statistical analysis	10	(8.1)
Pharmaceutical professional	6	(4.8)
Others[1]	9	(7.3)
**Professional title***		
Professor/Clinical Professor, or above	58	(46.8)
Associate Professor/Associate Clinical Professor, or relevant	28	(22.6)
Assistant Professor/Assistant Clinical Professor, or relevant	12	(9.7)
Lecturer, or relevant	12	(9.7)
Postdoc Fellow, or relevant	1	(0.8)
Research Assistant, or relevant	8	(6.5)
Students	3	(2.4)
Others[2]	2	(1.6)
**Types of Institutions***		
University	77	(62.1)
Hospital	42	(33.9)
Research institute	20	(16.1)
Journal agency	5	(4.0)
Regulatory agency	2	(1.6)
Industry/Company	2	(1.6)
Individual	3	(2.4)
**Geographical distribution**		
Mainland of China	85	(68.5)
South Korea	8	(6.5)
Australia	6	(4.8)
US	6	(4.8)

HKSAR, China	5	(4.0)
UK	4	(3.2)
Japan	2	(1.6)
Canada	2	(1.6)
Norway	1	(0.8)
Netherlands	1	(0.8)
Germany	1	(0.8)
France	1	(0.8)
Congo	1	(0.8)
Brazil	1	(0.8)

* Multiple choices for each participant, thus the total number is more than 124.

1 Including Not specified (3), Researchers (2), Teacher of Traditional Medicine (1), Clinician in integrative medicine (1), Biochemist (1), Clinician in epidemiology and pharmacology (1).

2 Including Executive manager (1), Not specified (1).

S5. The timeline for the implementation of this study.

Timeline	Implementations
December 2018 - March 2020	Identifying the necessity for this study of multicenter clinical trials through reviewing relevant studies, conducting preliminary investigations, seeking professional consultation, and facilitating discussions among the working group.
April 2020 - September 2021	Preparing and submitting the proposal of this study, obtaining research ethical approval, applying for grant and revising the proposal as needed, and ultimately launching the research project after funding received.
October 2021 - December 2022	Completing all necessary literature reviews, the Delphi survey, and the formulation of checklists for both the CONSORT and the SPIRIT extensions for multicenter clinical trials.
January 2023 - December 2023	Completing the pilot test, consensus meeting, and finalizing two Guideline statements with E&E documents.
January 2024 - June 2024	Completing the guideline publications, submitting the project report, and conducting disseminations based on planned strategies.
